# Abiotic Degradation of the Toxin Simplexin by Soil Collected from a *Pimelea*-Infested Paddock

**DOI:** 10.3390/toxins17030124

**Published:** 2025-03-06

**Authors:** Zhi Hung Loh, Natasha L. Hungerford, Diane Ouwerkerk, Athol V. Klieve, Mary T. Fletcher

**Affiliations:** 1 Queensland Alliance for Agriculture and Food Innovation (QAAFI), The University of Queensland, Health and Food Sciences Precinct, Coopers Plains, QLD 4108, Australia; zhihung.loh@uq.edu.au (Z.H.L.); a.klieve@uq.edu.au (A.V.K.); 2 Agri-Science Queensland, Department of Primary Industries (DPI), Ecosciences Precinct, Dutton Park, QLD 4102, Australia; diane.ouwerkerk@daf.qld.gov.au

**Keywords:** simplexin, *Pimelea*, hydrolysis, plant toxin, mass spectrometry

## Abstract

*Pimelea* poisoning of cattle is caused by the toxin simplexin present in native *Pimelea* plant species. Surface weathering and burial of *Pimelea* plant material under soil in *Pimelea*-infested pastures previously showed simplexin degradation, suggesting soil microbial metabolism and/or abiotic degradation of simplexin in the field. This current study investigated whether soil from a *Pimelea*-infested paddock was capable of simplexin degradation in the laboratory. The effects of temperature on isolated simplexin levels and simplexin levels in *Pimelea* plant material treated with field-collected soil, acid-washed sand or bentonite were determined. *Pimelea* plant material incubated in field-collected soil at 22 °C for seven days did not show any simplexin degradation. Isolated simplexin preadsorbed to field-collected soil, acid-washed sand or bentonite showed simplexin decrease after one hour of incubation at 100 °C with three breakdown products identified by UPLC-MS/MS, indicating that toxin breakdown can be a heat-induced process rather than a microbial-based metabolism. Decreased simplexin levels were observed in *Pimelea* plant material mixed with acid-washed sand under similar incubation conditions. Overall, the study showed the field-collected soil did not contain soil microorganisms capable of simplexin metabolism within a short period of time. However, the co-exposure to high temperature resulted in significant abiotic simplexin breakdown, without microorganism involvement, with the product structures suggesting that the degradation was a heat promoted acid hydrolysis/elimination process. Overall, this study demonstrated that simplexin breakdown in the field could be a thermal abiotic process with no indication of microbial involvement.

## 1. Introduction

**Figure 1:**
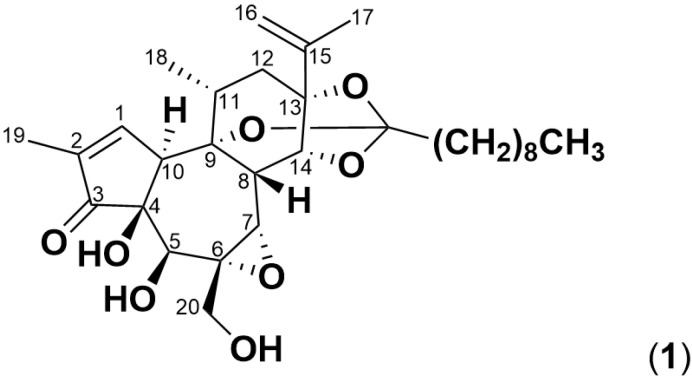
Structure of simplexin (**1**), a daphnane orthoester with a C9 saturated fatty acid chain.

*Pimelea* poisoning in cattle is attributed to three toxic plant species, which are *Pimelea trichostachya*, *P. simplex* and *P. elongata,* from which simplexin ((**1**), Figure 1) was identified to be the responsible toxin [1,2,3,4]. The poisoning is caused by the inadvertent consumption of *Pimelea* plant material by cattle during grazing, leading to the prolonged intake of small doses of plant material over time, with as little as 12.5 mg of *Pimelea*/kg bw/day demonstrated to induce poisoning [5]. The toxin targets the protein kinase C (PKC) present in the pulmonary venule and heart muscle of cattle resulting in the characteristic subcutaneous oedema observed at the brisket and head of cattle [1,3].

*Pimelea* species are native plants growing in arid rangeland regions of Australia where each of the plant species are found distributed in specific types of soils. *P. trichostachya* is found on sandy soils and duplex soils, *P. simplex* can be found on clay soils and red desert loams while *P. elongata* grows on loams, sandy red earth and claypans [1]. Surface weathering and burial of *Pimelea* plant material under soil in *Pimelea* affected pastures over 12 months showed simplexin degradation where simplexin was reduced by 50% within six months and less than 10% simplexin remaining after 12 months [1,6]. Simplexin was also not detected in soil that was in contact with the *Pimelea* plant unless the actual plant fragments remained in the soil as simplexin is hydrophobic in nature which prevents the toxin leaching into the soil [1]. Surface weathering of *Pimelea* plant material on top of the soil also showed higher simplexin loss compared to plant material that was buried under the soil [1]. The weathering study suggested that temperature and/or sunlight may be key factors in simplexin degradation in the field but does not exclude the involvement of soil microorganisms in simplexin metabolism.

**Figure 2:**
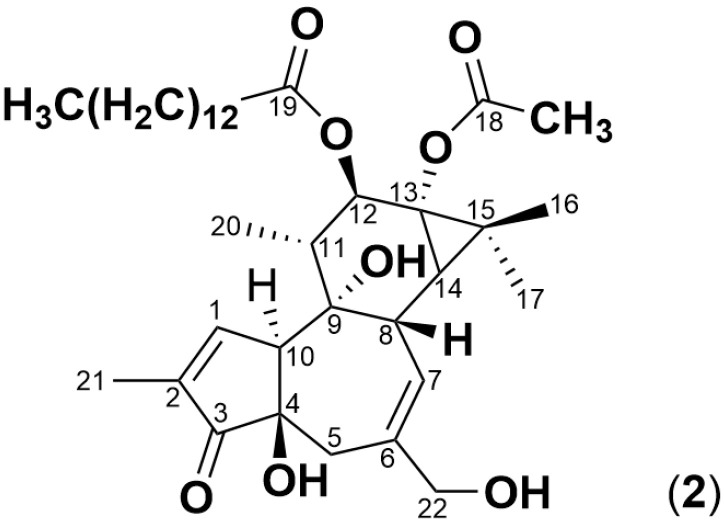
Structure of phorbol 12-myristate 13-acetate (**2**), a tetracyclic diterpenoid with two hydroxyl groups on neighboring carbon atoms esterified to a fatty acid/acetic acid.

Mycotoxins such as aflatoxin B_1_, deoxynivalenol and zearalenone have been reported to undergo degradation by soil microorganisms either in mixed culture form or in isolated form [7,8,9,10,11]. Pesticides of various classes were degraded by soil microorganisms which showed adaptation towards the pesticides after their application over time. A *Bacillus* soil bacterium strain was found to degrade profenofos, an organophosphorus pesticide, by up to 93% within 30 days where the bacterium was isolated from soil that was exposed to profenofos over a long period of time [12]. Another soil bacterium, isolated from soil exposed to acetamiprid for 10 years, was able to degrade acetamiprid in various contaminated soils and water sources within 48 h of incubation [13]. Devappa et al. [14] reported the biodegradation of phorbol esters of *Jatropha curcas* using soil that had no prior exposure to Jatropha plants. Phorbol esters such as phorbol 12-myristate 13-acetate (PMA, Figure 2) are tigliane diterpenoids [15] that have a similar skeletal structure to simplexin and are also PKC activators. These studies [7,8,9,12,13,14] confirmed the possibility of microbial degradation of toxins in soil.

To date, there is no preventative treatment against *Pimelea* poisoning other than management strategies based on the current knowledge of *Pimelea* and simplexin. As a means to develop a preventative treatment, attempts were made to identify rumen microorganisms capable of simplexin degradation from *Pimelea*-resistant ruminants, but no such rumen microorganisms were identified [16]. Therefore, alternative sources of microbes were investigated for simplexin degradation that could potentially be developed into a preventative probiotic against *Pimelea* poisoning. Manipulation of rumen microbiome has shown promise in degradation of other plant toxins as a poisoning mitigation strategy [17].

**Figure 3:**
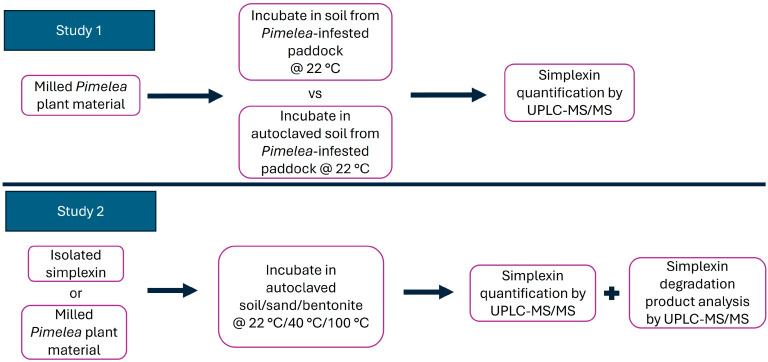
Summary of experiments using milled *Pimelea* plant material and isolated simplexin, with soil, sand and bentonite.

Currently, there is limited knowledge of simplexin degradation by soil microorganisms or of the effect of temperature, although the previous field study did seemingly indicate degradation in the field [1]. This study aimed to determine whether simplexin in *P. trichostachya* plant material can be degraded in soil collected from *Pimelea*-infested paddocks and whether soil microbes capable of simplexin degradation were present. The study also aimed to investigate the effect of temperature on isolated simplexin and simplexin in *Pimelea* plant material when incubated with field-collected soil and other materials. A summary of the experiments undertaken is depicted in Figure 3. Both *P. trichostachya* plant material and soil collected from *Pimelea*-infested paddocks were obtained from infested regions of southern inland Queensland [18].

## 2. Results and Discussion

### 2.1. Incubation of Milled P. trichostachya with Field-Collected Soil

Simplexin quantification on ultra-performance liquid chromatography-tandem mass spectroscopy (UPLC-MS/MS) was performed with isolated simplexin (**1**) as external standards and with PMA (**2**) as an internal standard to compensate for matrix effects in complex sample matrices. PMA (**2**) was used as internal standard since it is commercially available and has near identical tricyclic skeleton structure to simplexin. Both PMA (**2**) and simplexin (**1**) also have a long fatty acid side chain, albeit in diester and orthoester form respectively. This makes both compounds hydrophobic in nature and with closely similar retention times. Ideally, the internal standard should be an isotopic analogue of an analyte which ionises in the same manner, but there was no simplexin analogue available for this study.

Milled aerial parts of *P. trichostachya* mixed with field-collected soil showed no significant change in simplexin concentration during a seven-day incubation period at ambient temperature (22 °C) when compared to the sterile soil at ambient temperature (Table 1) suggesting simplexin degradation by soil microbes did not readily occur.

The microbial metabolism of phytochemicals can be induced in soil by various factors including microbial population composition in soil, soil physicochemical properties, moisture levels, light levels and temperature [14,19]. This initial incubation study was carried out in the dark and at room temperature to minimise any effect of light and temperature on simplexin degradation. Field-collected soil was also adjusted to a standard optimal 20% soil moisture prior to the incubation study to promote optimal microbial growth and activity [20,21]. Previous studies showed that a longer incubation period may be required for toxin degradation to occur in soil. The *Pimelea* weathering trial by Fletcher et al. [6] reported a simplexin reduction of 50% after six months. Slow degradation (26.7%) during the initial six days followed by high degradation (89.2%) by the ninth incubation day was observed for related phorbol esters in Jatropha seed cake mixed in soil at room temperature [14]. Soil incubation of the methyl ester of fusaric acid showed 50% degradation at day 20 on average when incubated at 20 °C with soil moisture kept at 75% field capacity [22]. The current soil degradation study had a one-week (168 h) incubation period to promote soil microorganisms capable of simplexin degradation. The one-week duration was chosen in order to select for microorganisms that can degrade simplexin within a short time period (akin to the digestion transit time of the cattle rumen [23]), with the aim being a microbial inoculum against simplexin.

The present incubation result (Table 1) suggests that there was no soil microbe present that was capable of simplexin metabolism within seven days and certainly not within 24 h. The absence of microbial degradation could be due to soil microbes requiring time to first adapt for utilising the toxin as a carbon source. The incubation also showed an apparent gradual albeit not significant increase of simplexin extracted from the plant over time during incubation. The apparent slight increase of simplexin extracted from the plant could be due to the variation of simplexin content in the milled plant samples added into each culture glass tube, however the greater than expected variation could likely reflect a greater ease of simplexin extraction from the plant material across days as the plant material was decomposed by microorganisms present in the plant.

The current results showed that the field-collected soil did not contain microorganisms capable of degrading simplexin in milled plant material within seven days, at least not under the experimental conditions that were imposed. Therefore, other variables needed to be considered for soil collected from *Pimelea*-infested paddocks to induce simplexin degradation, with these studies exploring both the degradation of isolated (purified) simplexin and also simplexin within *Pimelea* plant material. These studies were designed to determine whether delayed release of the toxin from plant material was a contributing factor in the observed stability of simplexin over prolonged periods in *Pimelea* field weathering studies [1,6]. Soil incubated with isolated simplexin may be able to select for soil microbes capable of simplexin degradation. It was previously shown that an *Arthobacter* sp. strain isolated from material from greened potatoes and the surrounding soils was identified to have the ability to degrade toxic glycoalkaloids α-chaconine and α-solanine when incubated with enrichment cultures for 20 h using minimal media containing both glycoalkaloids in purified form [24,25].

### 2.2. Simplexin Degradation by Heat

#### 2.2.1. Heating Effect on Isolated Simplexin Preadsorbed to Sand, Bentonite and Field-Collected Soil

As soil microbial metabolism of simplexin in *Pimelea* plant was not apparent, an additional approach using isolated simplexin placed in direct contact with the field-collected soil was attempted. Acid-washed sand was proposed as a binding agent for isolated simplexin to be mixed with field-collected soil for incubation. The sand-bound simplexin was subjected to steam sterilisation (121 °C, 30 min) prior to the incubation step to eliminate any microbes present so only soil microbes would be responsible for simplexin degradation. After sterilisation however, the simplexin concentration in sand-bound sample was observed to be considerably reduced. By comparison, simplexin that was added into aqueous media remained stable after the media was boiled and autoclaved at 105 °C for 45 min. Simplexin concentration in media (4274 ng/mL) were within the expected concentration of 6000 ng/mL with minimal simplexin loss possibly occurring at each media preparation step [26]. Isolated pure simplexin (expected concentration 1000 ng/mL) when heated alone at 100 °C for 1 h was also found to remain unchanged (1017 ng/mL) [26]. Therefore, a further study was carried out to investigate the effect of temperature on isolated simplexin adsorbed to different materials (acid-washed sand, sodium bentonite and field-collected soil).

**Figure 4:**
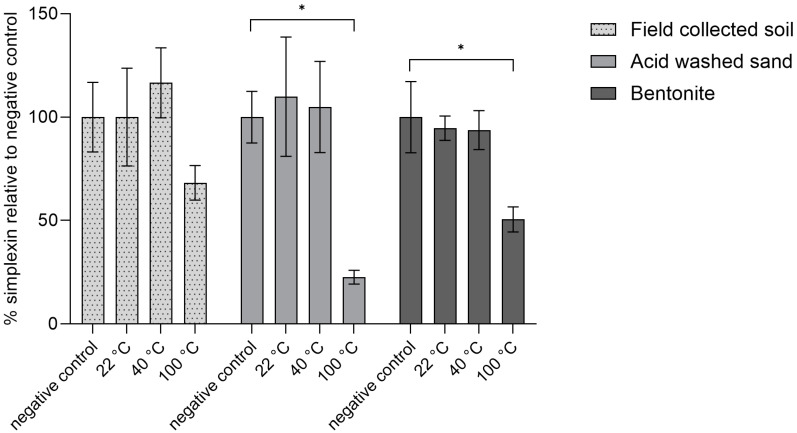
Percentage isolated simplexin decrease for each autoclaved binding agent (*n* = 3) after heat treatment for 1 h relative to negative control (100%). Significant differences between treatment groups vs. negative controls (no heat treatment) are indicated by asterisks (*p* < 0.05).

In the current study, sterilised (autoclaved) field-collected soil, sand or bentonite were used as binding agents for simplexin and incubated for one hour at room temperature (22 °C), at 40 °C simulating the expected average soil temperature in the field and cattle rumen temperature, and at 100 °C to determine the stability of simplexin with each binding agent at a higher temperature. The simplexin extraction of field-collected soil, sand and bentonite negative control samples (not heated and immediately extracted, Figure 4) was used to determine the percentage degradation at each temperature. No significant extracted simplexin decrease was observed for all materials incubated with simplexin at room temperature (22 °C) or at 40 °C. At 100 °C, significant loss of simplexin was observed in sand (77% decrease, *p* = 0.005) and bentonite (49% decrease, *p* = 0.002) samples when compared to their respective negative control (Figure 4). Isolated simplexin bound to field-collected soil after incubation at 100 °C appeared to show evidence of a decrease in simplexin level (31%) when compared to its negative control (not significant, *p* = 0.191) (Figure 4).

The current results showed significant simplexin decrease only occurred in simplexin incubated at 100 °C when compared to the negative control for each sterilised material. Physical treatment by dry heating at high temperature is a common detoxification strategy for a wide range of mycotoxins and toxic proteins removing them from feed prior to consumption [27,28,29]. Similarly, silica-bound phorbol esters were reported to undergo faster initial rates of degradation at higher temperature (32 °C and 42 °C) compared to room temperature (23 °C) [14]. Phytotoxins (ptaquiloside and caudatoside) from bracken were found to undergo abiotic removal (not detected within 24 h) after incubation with autoclaved filter sands collected from waterworks for 14 days at 8–10 °C in the dark [30]. The results in the current study suggest that material-bound simplexin undergoes rapid degradation at high temperature (100 °C).

After 100 °C incubation, sand-bound simplexin was observed to have the largest simplexin decrease followed by bentonite-bound simplexin and soil-bound simplexin. Sand is made up of silica that can act as an oxidative catalyst [31] and the acid-washed sand purchased for this study contained up to 0.2% soluble HCl matter (as per product certification) which could also act as an acid catalyst. The purchased bentonite consists of mostly smectite clay which has been reported to be an acid catalyst [32,33]. Degradation of *J. curcas* phorbol esters by soil was reported to depend on the temperature, pH, adsorption capacity, clay mineralogy, organic matter content and soil particle size, as well as compound properties and microbial populations [14]. The *Pimelea* soil collected in this study was observed to be a sandy duplex soil as is reported being commonly associated with *P. trichostachya* plant found around the collection area [1]. Sandy duplex soils in general contain varying degrees of sand and clay on their surface, depending on the location, which influences the acidity of the soil [34]. In the present study, simplexin bound in these materials was postulated to undergo acid catalysed breakdown in the presence of high temperature. Aqueous simplexin hydrolysis using acid as a catalyst was previously reported in Loh et al. [35] and by Freeman et al. [36] under reflux conditions.

Overall, isolated simplexin showed degradation after one hour of incubation at 100 °C with the pre-absorbed sand having the highest simplexin degradation followed by bentonite and field-collected soil (Figure 4). The simplexin breakdown products in each sample were identified by UPLC-MS/MS and will be further discussed below (Section 2.3).

#### 2.2.2. Heating Effect on Milled *P. trichostachya* Mixed with Field-Collected Soil, Sand and Bentonite

**Figure 5:**
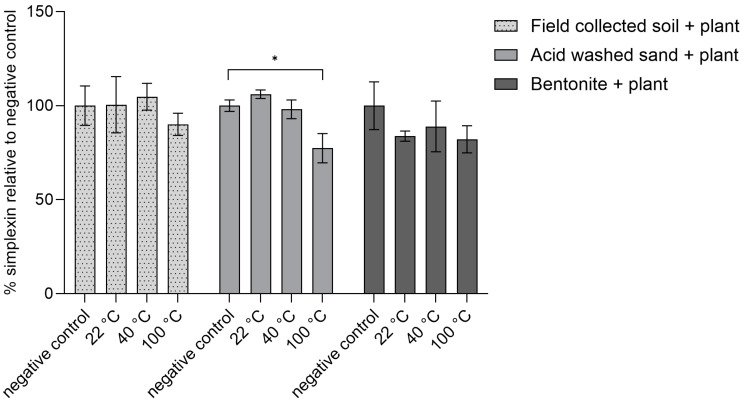
Percentage simplexin decrease in *Pimelea* plant material (*n* = 3) for autoclaved soil, sand and bentonite materials after heat treatment for 1 h relative to negative control (100%). Significant differences between treatment groups vs. negative controls (no heat treatment) are indicated by asterisks (*p* < 0.05).

As isolated simplexin showed degradation at higher temperature (100 °C) in the presence of each sterilized material, another study was conducted to observe whether simplexin within milled *Pimelea* plant could be degraded by sterilized field-collected soil, sand and bentonite under the same temperature conditions and the same incubation time. Plant material mixed in sand showed significant decrease in simplexin (23%) after incubation at 100 °C while no significant decrease in simplexin was observed for the room temperature (22 °C) and 40 °C incubated sand samples when compared to their respective negative control (Figure 5). Hence, heat was an important promoter of the degradation of simplexin. Plant material mixed with field-collected soil and bentonite incubated at all temperatures did not show any significant simplexin degradation.

Fletcher et al. [1] noted that *P. trichostachya* litter buried under 1–2 cm of soil maintained higher residual simplexin than *P. trichostachya* litter left on top of the soil at the end of the 12 months field weathering trial study and the authors proposed that temperature and/or sunlight could play an important role in simplexin breakdown. In the present study, simplexin in plant material mixed with acid-washed sand at 100 °C (Figure 5) was thought to have been transformed in a manner similar to the sand-bound isolated simplexin sample above (Figure 4). However, simplexin in *Pimelea* plant material mixed with field-collected soil and bentonite at 100 °C did not result in significant simplexin degradation. The present study also showed degradation of simplexin in plant material mixed with sand (23%) was lower compared to the degradation of isolated simplexin bound to sand (77%). Isolated simplexin was more likely to be directly in contact with abiotic agents such as acid and moisture to allow degradation to occur when heated. Simplexin in the plant, however, was required to be released from the plant material first before the degradation reaction could occur.

The study suggested that simplexin in the plant was protected and needed to be released from the plant first before degradation could occur. Only plant mixed with acid-washed sand showed significant simplexin degradation within one hour of incubation at 100 °C.

### 2.3. Simplexin Breakdown Products

**Figure 6:**
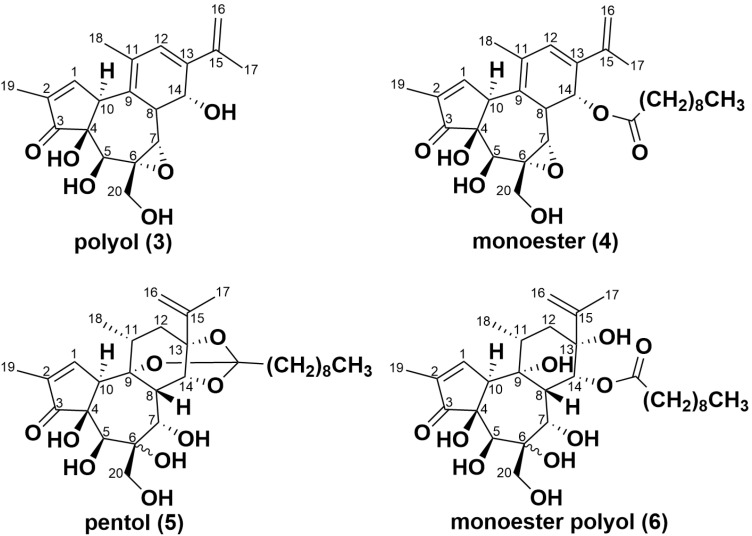
Proposed chemical structures of ring-opened simplexin breakdown products (**3**), (**4**) and (**5)** corresponding to their respective identified molecular formulae based on HRAMS, with stereochemistry and regiochemistry of the C6/C7 in (**5**) not determined. Monoester polyol (**6**) although observed previously in acid hydrolysis studies [35] was not detected in the current degradation studies.

**Figure 7:**
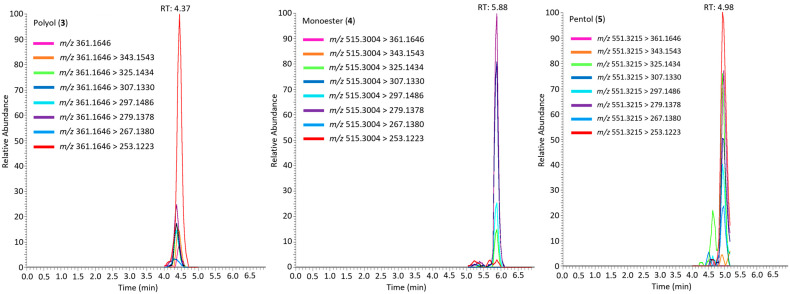
Extracted ion chromatogram of simplexin degradation products (**3**), (**4**) and (**5**) in field-collected soil bound simplexin after one hour incubation at 100 °C showing their transitions in positive ionisation mode using typical simplexin fragment ions. Similar chromatogram patterns were observed for simplexin similarly treated with acid-washed sand and bentonite samples.

Extracts from simplexin bound with sterilised field-collected soil, acid-washed sand and bentonite incubated at 100 °C were also analysed to identify simplexin breakdown products using UPLC-MS/MS. Three simplexin breakdown products (Figure 6) were identified based on HRAMS corresponding to molecular formulae C_20_H_24_O_6_, C_30_H_42_O_7_ and C_30_H_46_O_9_ (Figure 7) and were given the trivial names polyol (**3**), monoester (**4**) and pentol (**5**) respectively. These products were not observed during UPLC-MS/MS analysis of the extracts from the negative control experiments. The mass spectra of the breakdown products showed strong intense [M + H]^+^ adduct peaks in positive ionisation mode. The incubated degradation products had molecular formulae matching the H_2_SO_4_ catalysed hydrolysis/elimination products described in Loh et al. [35], with their formation in this instance promoted by heat. However, the H_2_SO_4_ hydrolysis product named monoester polyol (**6**) reported in Loh et al. [35] was absent in this study in extracts from simplexin bound with sterilised field-collected soil, acid-washed sand and bentonite incubated at 100 °C samples.

Fragment ions *m/z* 343, 325, 307, 297, 279, 267 and 253 of breakdown products (**3**)–(**5**) matched those observed for simplexin and H_2_SO_4_ hydrolysis products reported in Loh et al. [35] and each ion was within 5 ppm of the calculated accurate masses. All seven selected fragment ions of breakdown product (**4**) had matching ion ratios to the monoester identified from aqueous H_2_SO_4_-mediated hydrolysis of simplexin suggesting that both products were identical [35].

On the other hand, the relative intensities of shared fragment ions of breakdown products (**3**) and (**5**) differed from the polyol and pentol identified from H_2_SO_4_ catalysed hydrolysis of simplexin. In breakdown product (**3**), fragment ion *m/z* 253 had the highest intensity followed by *m/z* 279 while the polyol from H_2_SO_4_ hydrolysed simplexin showed the two highest fragment ion intensities to be *m/z* 253 followed by *m/z* 267 [35] (as for simplexin). Breakdown product (**5**) showed the highest fragment ion intensity at *m/z* 253 with the second highest fragment ion intensity to be *m/z* 325 and 297. By comparison, the pentol from the H_2_SO_4_-mediated hydrolysis of simplexin showed *m/z* 279 as the highest intensity fragment ion, followed by *m/z* 253 [35]. The difference in fragment ion ratios between the breakdown products in the current study and the previous simplexin H_2_SO_4_ hydrolysis suggests different reaction pathways had occurred resulting in different regiochemistry or stereochemistry of the breakdown products. The reaction in this study involved solid reaction conditions while the reaction in Loh et al. [35] occurred in aqueous solution. Therefore, different breakdown products could have formed during the reactions, but due to the highly dehydrated nature of the fragment ions mentioned, little further information can be gleaned from them.

None of the breakdown products (**3**)–(**6**) could be detected in heat-treated milled *Pimelea* plant material samples mixed with sterilised sand. This could be due to the reduced rate of simplexin degradation in plant material (23%), such that ions of breakdown products were below the limit of detection of the instrument or alternately that the initial hydrolysis products were degraded further within the plant sample.

## 3. Conclusions

Overall, this laboratory study suggests that there is no microbial involvement in simplexin breakdown, which instead involves a thermal abiotic process, with likely implications for what occurs in the field. In-laboratory experiments mixing soil collected from a *Pimelea*-infested paddock with milled *Pimelea* plant material did not result in the microbial metabolism of simplexin, suggesting the soil did not have microbes that were capable of simplexin degradation within a biologically relevant time frame. Simplexin also could have low bioavailability to the soil microbes due to its hydrophobic nature, known to limit transport across the cell membrane of the microbes [37]. Further investigation on the effect of temperature on isolated simplexin adsorbed to sterilised acid-washed sand, bentonite and field-collected soil showed simplexin levels decreased significantly after one hour incubation at 100 °C. A similar simplexin decrease was observed in acid-washed sand mixed with milled *Pimelea* plant material heated to 100 °C for one hour. Three simplexin breakdown products were identified in simplexin preadsorbed to materials with predicted structures suggesting the degradation was a heat promoted acid hydrolysis/elimination process. No simplexin degradation, however, was observed with simplexin in isolated form and in plant material that was incubated for one hour with field-collected soil at 40 °C comparable with the highest daily maximum air temperature of the collection location [38], but it is expected that such degradation could occur over longer time periods as transpires in the field [1]. These results suggest that in the short-term toxin breakdown can be an abiotic heat-induced degradation process rather than a microbial-based metabolism.

Further investigations are required to determine the ability of the materials to degrade isolated simplexin at 40 °C for a longer incubation time, typical of field conditions. A longer incubation time would also help to determine the ability of the materials to degrade simplexin in plants at 40 °C and 100 °C, and whether the same breakdown products identified from pure simplexin are also formed. This would allow further understanding of the time required for simplexin to be released from the plant before degradation or metabolism in the soil could occur. Degraded simplexin compounds have yet to be isolated for structural elucidation and toxicity determinations which would require much larger-scale experiments. The availability of these degradation products would enable further studies to investigate their potential further degradation either abiotic or microbial.

## 4. Materials and Methods

### 4.1. General Experimental Materials

Aerial parts of *Pimelea* plant were milled using a Christy and Norris 8000 RPM 8” Laboratory Mill (Ipswich, UK) fitted with a 3 mm screen. For simplexin isolation from *Pimelea*, plant extract was shaken on a Model RP1812 reciprocating shaker (Paton Scientific, Victor Harbour, SA, Australia) set at 200 rpm. Extracts were sonicated on an Elma Transsonic Digital S ultra-sonic water bath (Elma, Singen, Germany) set to 140% ultrasound power and 40 kHz ultrasound frequency. Crude *Pimelea* extracts were filtered using a glass-fibre filter paper (Whatman, Merck, Castle Hill, Australia). Extracts were concentrated by solvent removal under reduced pressure using a Buchi R-200 rotavapor system (Buchi, Flawil, Switzerland). For simplexin isolation from plant crude extract, flash chromatography was performed using silica gel 60 (0.040–0.063 mm) for column chromatography (230–400 mesh ASTM) (Merck, Castle Hill, NSW, Australia), under a positive pressure of nitrogen. Analytical thin-layer chromatography (TLC) was performed on TLC silica gel 60 F_254_ (Merck, Castle Hill, NSW, Australia). Visualisation was achieved by ultraviolet light at 254 nm and alternatively a red-brown spot developed using a reagent consisting of ammonium metavanadate (4 g) dissolved in 50% sulphuric acid (200 mL) prepared as described in Sakata et al. [39]. All TLC analyses were performed as above unless stated otherwise.

Sample incubation at 40 °C was performed by heating a metal heat block on a magnetic stirrer with heating plate (IKA^®^ C-MAG HS 4, Staufen, Germany) to 40 °C and the temperature was monitored using a thermometer attached to the metal heat block. Sample incubation at 100 °C was completed in a dehydrating oven (Thermoline Scientific, Wetherill Park, NSW, Australia). Acid-washed sand (Ajax Finechem, Merck, Castle Hill, NSW, Australia) was purchased for the sand incubation study. Bentonite (Sibelco Trufeed Sodium Bentonite Clay (Sodium Bentonite), purchased from Landmark, St. George, Australia), was ground with a mortar and pestle to produce a uniform material for labscale experiments. Materials were autoclaved (121 °C, 30 min) using a Model T62 autoclave (Sabac, Yeronga, QLD, Australia). The same rotavapor, reciprocating shaker and centrifuge were used for sample preparation and simplexin extraction.

The internal standard phorbol 12-myristate 13-acetate (PMA (**2**), also known as 12-*O*-tetradecanoyl-phorbol-13-acetate) was purchased from Merck (Castle Hill, NSW, Australia). Solvent compositions were mixed *v*/*v* as specified. ChemDraw Professional version 19.0 (PerkinElmer, Waltham, MA, USA) was used to draw all chemical structures and generate molecular formulae.

### 4.2. Sample Material Collection for the Degradation Study

#### 4.2.1. Soil

Field-collected soil from the top 0–10 cm layer used in the incubation studies was collected from a paddock where *Pimelea* was growing on a property near Wandoan, Queensland in September 2020 with collection coordinate noted (−26.16299, 149.98296). The soil was stored in buckets sealed with lids and transported to the QAAFI Natural Toxins Laboratory at Coopers Plains. Soil material was sieved (mesh size 1 mm) and homogenised to ensure soil was free of any extraneous plant material. Field-collected soil was then stored in a bucket with lid closed at room temperature until further use.

#### 4.2.2. Plant Material

*Pimelea trichostachya* plants (~30 kg dried plant weight) used in this study were collected from a property near Bollon, Queensland, with collection coordinate noted (−27.57023, 147.686327) in October 2020. Plant identification was confirmed by Queensland Herbarium and botanical specimens were incorporated into their permanent collection as vouchers (AQ952584). Collected plants were air dried at the DAF Charleville facility and then transported to the QAAFI Natural Toxins Laboratory at Coopers Plains. Plant parts were separated and the composite aerial portion which included seeds, flower heads, small stems and leaves were milled. Milled plant materials were stored frozen at −40 °C until further use. Simplexin quantification by UPLC-MS/MS using isolated simplexin as external standard (no internal standard) was performed as described in Loh et al. [35]. The milled plant materials were quantified to contain 103 mg/kg simplexin [16].

### 4.3. P. trichostachya Seed Collection and Simplexin Isolation from Seeds

Seed material was collected for the isolation of simplexin, which was used for the simplexin recovery study, as a calibration standard and for adsorption to materials used in soil spiking experiments. Seed material was obtained from *P. trichostachya* collected from a property near St George, Queensland in September 2018 with collection coordinate noted (−27.98, 148.18575). Plant identification was confirmed by the Queensland Herbarium with the botanical specimen incorporated into their permanent collection as a voucher (AQ522769). Field-collected plant samples were transported to the QAAFI Natural Toxins Laboratory in Coopers Plains and air-dried before seed materials were separated from the plant. The seeds were milled and kept frozen (−40 °C) until extraction.

Simplexin isolation was performed as previously reported in Chow et al. [2]. In short, milled seeds of *P. trichostachya* (121.54 g) were extracted twice using 90% methanol. The methanol extracts were combined and concentrated under reduced pressure. The concentrated extract was further extracted by liquid-liquid extraction using saturated sodium chloride solution and dichloromethane with the dichloromethane extract collected, dried and evaporated under reduced pressure. The residue was redissolved in hexane and extracted with acetonitrile. The acetonitrile extract was evaporated under reduced pressure and analysed by TLC to observe for red-brown spot on standing. The residue was applied to flash chromatography and fractions containing simplexin were combined, concentrated and subjected to further flash chromatography. Fractions containing simplexin were identified using TLC and UPLC-MS/MS, combined and concentrated to give crude simplexin (0.22 g). The crude simplexin residue was purified by preparative HPLC using protocol described in Loh et al. [35]. Elution of simplexin was guided using previously isolated and purified simplexin. Eluted simplexin was collected into one fraction, concentrated under reduced pressure and freeze-dried overnight to produce solid yellow simplexin crystals (126.9 mg, 0.1% yield from seed material, 95% purity, confirmed by NMR and UPLC-MS/MS analyses). Purified simplexin (**1**) was used for incubations in Section 4.5.1 and as external standard in Section 4.6.

### 4.4. Incubation of Milled P. trichostachya on Field-Collected Soil

Field-collected soil samples (0.5 g) were placed in 15 mL culture test tubes with screw caps and adjusted to 20% soil moisture (initial soil moisture of field-collected soil stored in 22 °C was 0%) on dry weight basis using distilled water. Six sets of triplicate soil samples were prepared for collection at time intervals of 0, 1, 2, 3, 6 and 7 days. Samples were then incubated in the dark at ambient laboratory temperature (22 °C) for seven days to allow the growth of soil microbes. After seven days, milled *P. trichostachya* (0.1 g, Section 4.2.2) was mixed with the field-collected soil and incubated in the dark at ambient laboratory temperature (22 °C) for one week. Glass tubes were collected at the determined time intervals. Six sets of triplicate negative controls were also prepared by autoclaving (121 °C, 30 min) field-collected soil first and then proceeding as for the non-sterile soil samples.

After incubation, samples were transferred into 15 mL centrifuge tubes, methanol (2 mL) was added, and then the samples were shaken for one hour for simplexin extraction. Samples were centrifuged in an Eppendorf 5810 centrifuge (Eppendorf, Hamburg, Germany) at 3203× *g* for 5 min and the supernatants collected into fresh 15 mL centrifuge tubes. Pellets were re-extracted twice more with methanol (2 × 2 mL), shaken, centrifuged with the supernatant added to respective labelled centrifuge tubes giving a total volume of 6 mL each. A portion of the sample extract (1 mL) was mixed with internal standard PMA ((**2**), 50 µL, 1 µg/mL in methanol) and filtered via 0.2 µm membrane syringe filters (GHP Acrodisc, Pall, Cheltenham, Australia) into MS vials for UPLC-MS/MS analysis (Section 4.6). Simplexin concentrations were back calculated with the amount of milled *P. trichostachya* added into the soil to give the simplexin amount per kg of plant material.

### 4.5. Simplexin Degradation by Heat

#### 4.5.1. Heating Effect of Isolated Simplexin Preadsorbed to Sand, Bentonite and Field-Collected Soil

Acid-washed sand, bentonite and field-collected soil were treated similarly. Each material (sand/bentonite/soil material) was autoclaved (121 °C, 30 min) first, and then pure isolated simplexin (Section 4.3) was adsorbed to the material. A simplexin preadsorbed stock sample was made for each autoclaved material by adding an aliquot (1 mL) of a simplexin solution (120 µg/mL) in methanol to the material (10 g). The mixture was thoroughly mixed, and methanol was removed under reduced pressure at 30 °C using a rotavapor to produce each solid material preadsorbed with simplexin (~12 mg/kg). For each preadsorbed material, triplicate samples (0.5 g) in culture test tubes with screw caps were maintained at ambient laboratory temperature (22 °C), at 40 °C or at 100 °C respectively, for one hour. For each material, a set of negative controls in triplicate was prepared from the same simplexin preadsorbed stock and was immediately extracted for simplexin without any temperature treatment.

After incubation, samples were extracted for simplexin and prepared for UPLC-MS/MS analysis as detailed in Section 4.4. Simplexin concentration was back-calculated with the amount of pure isolated simplexin preadsorbed to the materials to give the simplexin amount per kg of material.

#### 4.5.2. Heating Effect on Milled *P. trichostachya* Mixed with Field-Collected Soil, Sand and Bentonite

Acid-washed sand, bentonite and field-collected soil were treated similarly. For each material, sand/bentonite/soil materials were autoclaved (121 °C, 30 min) first as the material stock sample. Milled *P. trichostachya* (0.1 g, Section 4.2.2) was mixed with the material stock sample (0.5 g) in triplicate and placed into culture test tubes with screw caps. For each material, a set of negative controls in triplicate was also prepared from the same material stock sample, with *P. trichostachya* (0.1 g, Section 4.2.2) added followed by immediate extraction of simplexin without any temperature treatment. Samples were incubated as detailed in Section 4.5.1. Simplexin extraction and preparation for UPLC-MS/MS analysis were completed as described in Section 4.4. Simplexin concentration was back calculated with the amount of milled *P. trichostachya* added into the materials to give the simplexin amount per kg of plant material.

### 4.6. Simplexin Quantification in Plant and Preadsorbed Material Samples

Simplexin quantification in plant and preadsorbed material by UPLC-MS/MS utilised the method reported by Loh et al. [35]. In brief, simplexin was separated on a Waters Acquity UPLC BEH Shield RP_18_ column (2.1 mm × 100 mm, 1.7 µm) column with gradient elution. Simplexin detection was performed using targeted MS/MS in positive ionisation mode with parameters as described by Loh et al. [35]. MS/MS inclusion list was used, targeting protonated simplexin ([M + H]^+^, *m/z* 533.3109) and protonated PMA ([M + H]^+^, *m/z* 617.4080). Simplexin calibration curve (50–2000 ng/mL) was prepared using isolated simplexin in methanol (1 mL) followed by the addition of 50 µL of the internal standard PMA (1 µg/mL in methanol) before filtering via 0.2 µm membrane syringe filters (GHP Acrodisc, Pall, Cheltenham, VIC, Australia) into MS vials. MS/MS fragments analysis employed Thermo Xcalibur Qual Browser version 4.0.27.19 (ThermoFisher Scientific, Waltham, MA, USA). Simplexin quantification was performed using ThermoFisher TraceFinder version 4.1 (ThermoFisher Scientific, Waltham, MA, USA) utilising two major fragment ions of *m/z* 533.3109 > 253.1223 as transition of quantification and *m/z* 533.3109 > 267.1381 as transition of verification respectively. PMA (**2**) was identified using major fragment ion of *m/z* 617.4080 > 311.1642 used as the internal standard response peak for simplexin quantification.

### 4.7. Simplexin Degradation Product HRAMS Analysis

Sample separation was performed using the UPLC-MS/MS protocol as previously described in Section 4.6. MS detection was performed and operated in a similar mode as previously described in Section 4.6 with inclusion list adapted for simplexin degradation products (Table 2).

### 4.8. Statistical Analysis

All data were reported as mean ± SD. Graphs were produced using GraphPad Prism^®^ 9 (GraphPad Software, San Diego, CA, USA). One-way ANOVA followed by post hoc Tukey HSD analysis was performed using IBM SPSS Statistics 27 (IBM, Armonk, NY, USA) to compare the means. Data with *p* < 0.05 was considered statistically significant.

## Figures and Tables

**Table 1:** Simplexin concentration in milled aerial parts of *P. trichostachya* mixed with sterile and non-sterile field-collected soil at room temperature (22 °C) across one week of incubation.

	Simplexin Concentration (mg/kg Plant Material)					
	Day 0	Day 1	Day 2	Day 3	Day 6	Day 7
Sterile field-collected soil + *Pimelea*	85.3 ± 19.2 ^a^	96.4 ± 10.7 ^a^	101.1 ± 29.3 ^a^	100.1 ± 25.0 ^a^	104.2 ± 19.6 ^a^	117.1 ± 20.1 ^a^
Field-collected soil + *Pimelea*	72.8 ± 8.1 ^a^	99.4 ± 6.2 ^a^	98.5 ± 8.8 ^a^	100.7 ± 13.7 ^a^	94.6 ± 23.7 ^a^	103.5 ± 22.2 ^a^

Results were presented in mean ± SD (*n* = 3). Values followed by the same letters across days and soil vs. sterile soil were not statistically different (*p* > 0.05).

**Table 2:** Inclusion list for simplexin and simplexin degradation product UPLC-MS/MS analysis.

Molecular Formula	Species	Calculated Molecular ion (*m/z*) [M + H]^+^	Compound
C_30_H_44_O_8_	+ H^+^	533.3109	simplexin (**1**)
C_36_H_56_O_8_	+ H^+^	617.4080	PMA (**2**)
C_20_H_24_O_6_	+ H^+^	361.1646	polyol (**3**)
C_30_H_42_O_7_	+ H^+^	515.3003	monoester (**4**)
C_30_H_46_O_9_	+ H^+^	551.3215	pentol (**5**)
C_30_H_48_O_10_	+ H^+^	569.3320	monoester polyol (**6**)

## Data Availability

The original contributions presented in the study are included in the article, further inquiries can be directed to the corresponding authors.
